# The European Union Settlement Scheme Data Linkage Project in Wales: Findings relating to mental health and education

**DOI:** 10.23889/ijpds.v9i5.2567

**Published:** 2024-09-18

**Authors:** Ffion Lloyd-Williams, Matthew Curds, Stephen Drinkwater

**Affiliations:** 1 Welsh Government; 2 University of Roehampton

## Objective

Following the UK’s exit from the European Union (EU) a settlement scheme was introduced for EU nationals currently living in the UK to apply to continue living in the UK. The European Union (EU) Settlement Scheme project compares the living experiences of EU and UK citizens in Wales.

## Approach

We conducted a retrospective population cohort study within the Secure Anonymised information Linkage (SAIL) Databank to explore the mental health, educational experiences and involvement in the Welsh labour market of EU citizens living in Wales in comparison to British citizens. UK Census data relating to country of birth, sex, week of birth, date of arrival in the UK and socio-economic status have been linked to variables of relevance in the Education for Wales and Welsh General Practice datasets respectively and analysed.

## Results

Findings indicate statistically significant differences in secondary school attendance and attainment between Welsh born pupils and pupils born in EU countries. We will present findings on our exploration if differences exist between Welsh born and EU country born individuals in relation to diagnosis of depression and anxiety. Additional findings from this linking and analysis reveal other interesting differences between EU and UK born citizens.

## Conclusions

Linking data in this way has gained a better understanding of the experiences and outcomes of EU citizens in Wales, generating better evidence to inform policies and services that address the needs of this population and a dataset of interest to academics. Reproduceable analytical pipelines have been created for other researchers to use.

